# Identity blues: the ethnobotany of the indigo dyeing by Landian Yao (Iu Mien) in Yunnan, Southwest China

**DOI:** 10.1186/s13002-019-0289-0

**Published:** 2019-02-19

**Authors:** Shan Li, Anthony B. Cunningham, Ruyan Fan, Yuhua Wang

**Affiliations:** 1 Key Laboratory of Economic Plants and Biotechnology, Kunming Institute of Botany, Chinese Academy of Sciences, Kunming, 650201 China; 2 University of Chinese Academy of Sciences, Beijing, 100049 China; 3 School of Veterinary and Life Sciences, Murdoch University, 90 South St., Murdoch, WA 6150 Australia

**Keywords:** Landian Yao, Ethnobotany, Dye plant, Indigo dyeing

## Abstract

**Background:**

Indigo-dyed textiles have been central to the cultural identity of Landian Yao (literally “blue clothes Yao”) people in Southwest China for centuries, driving a significant local market for naturally dyed indigo cloth. In the past two decades, local indigo production for traditional textiles has declined for several reasons: Firstly, the younger generation of Landian Yao has shifted to using western style jeans and T-shirts. Secondly, due to its labor-intensive nature. In contrast, at a global scale, including in China, there has been a revival of interest in natural indigo use. This is due to a growing awareness in the fashion industry about human and environmental health issues related to synthetic dye production. Ironically, this new awareness comes at a time when traditional knowledge of indigo dyeing is being lost in many places in China, with weaving and use of natural dyes now limited to some remote areas. In this study, we recorded indigo dyeing processes used by Landian Yao people and documented the plant species used for indigo dyeing.

**Methods:**

Field surveys were conducted to the study area from September 2015 to November 2016, supplemented by follow-up visits in July 2018 and November 2018. We interviewed 46 key informants between 36 and 82 years old who still continued traditional indigo dyeing practices. Most were elderly people. Semi-structured interviews were used. During the field study, we kept a detailed account of the methods used by Landian Yao dyers. The data were then analyzed by using utilization frequency to determine the best traditional recipe of indigo dye extraction. All the specimens of documented species were collected and deposited at the herbarium of Kunming Institute of Botany.

**Results:**

Our results showed that indigo dyeing was divided into two main steps: (1) indigo pigment extraction and (2) dyeing cloth. The general procedures of indigo dye extraction included building or buying a dye vat, fermentation, removal of the leaves of indigo producing plant species, addition of lime, oxygenation, followed by collection, and the storage of the indigo paste. The procedures of dyeing cloth included preparing the dye solutions, dyeing cloth, washing, and air drying. It is notable that Landian Yao dyers formerly only performed the dyeing process on the goat days in the lunar calendar from June to October. After comparing the range of local indigo extraction methods, our results showed that the following was best of these traditional recipes: a indigo-yielding plant material to tap water ratio of 30 kg: 200 l, lime 3 kg, a fermentation time of 2–3 d, aeration by agitation for up to 60 min, and a precipitation time of 2–3 h. Our results show that 17 plant species in 11 families were recorded in the indigo dyeing process. With the exception of the indigo sources, only *Dioscorea cirrhosa* Lour. and *Artemisia argyi* H.Lév. & Vaniot were previously recorded in dyeing processes. Other species given in this paper are recorded for the first time in terms of their use in the indigo dyeing process. In the study area, Landian Yao men were in charge of indigo dye extraction, and the women were responsible for dyeing cloth.

**Conclusions:**

The Landian Yao has completely mastered the traditional indigo dyeing craft and are one of the well-deserved identity blues. Indigo production from plants using traditional methods is a slow process compared to synthetic dyes and is not suitable for modern and rapid industrial production. Therefore, our study records the detailed information of traditional indigo dyeing to protect and inherit it. *Strobilanthes cusia* (Nees) Kuntze is the main indigo source in Landian Yao that is widely used in the world and can be commercially exploited as an indigo plant. For commercial and environment benefits, we suggest that producing natural indigo for the commercial market is a good choice.

## Background

Blue colors are not uncommon in nature but can be difficult to extract. Because of its scarcity, indigo has been a valued and widely used dyestuff from prehistoric times. The earliest records of dyeing textiles with indigo dye originate from the 6000-year-old cotton fabrics discovered in the north coast of Peru, most likely from an *Indigofera* species native to South America [1]. The earliest written occurrence of indigo dye was probably in India ca. 2600 B.C. and it is also mentioned in Sanskrit writings from this time [2]. Evidence of the use of indigo dyes dates back to 2000 B.C. in ancient Egypt [3]. The earliest woad-dyed textiles in Europe to date have been found in the salt mine in Hallstatt, Austria and are up to 3500 years old [4]. The steel-gray cotton fabrics unearthed in Wuyi Mountain proved that indigo dyeing in China could be traced back to 3400 years ago [5]. In the course of the history of human civilizations, indigo dye is the only stable blue dye until the introduction of synthetic dyes in the late nineteenth century.

The primary limitations of extracting dyes or pigments from plant material are the relatively low color and light fastness and the time-consuming process [6]. Consequently, the wide range of colors available from synthetic dyes with good fastness properties and lower costs was the main reason of synthetic dyes substituted for natural dyes [7]. Synthetic indigo was obtained from aniline in 1878 [8]. Synthetically-produced indigo was of superior quality to indigo from plants and was therefore preferred by dyers [9]. Although some indigenous communities in remote areas continue to use indigo from plants, most industrially used indigo is synthetic [10].

Unfortunately, synthetic dyes have a number of important side effects on people and the environment. These include health and safety risks caused by toxic mordants and the high energy costs [11]. For example, sodium dithionite (Na_2_S_2_O_4_) is used as a reducing agent in modern indigo dyeing processes and this reducing agent and its derivatives are major pollutants of the textile industry and have negative effects on human health [10]. In recent years, it observed an increased interest in natural dyes because of the growing awareness of the toxicity and pollution resulting from the synthetic dyes [12–14]. As natural dyes are re-introduced into the market, natural indigo from plants is of great interest to environmentally and socially ethical clothing companies [15]. In addition, textiles dyed with natural indigo from plants have anti-bacterial activity when compared to synthetic indigo [16].

Throughout the world, every community has its own indigo dyeing methods which include different indigo-yielding plant species, recipes, techniques and rituals associated with traditional dye processes. Indigo dyes for traditional textiles have been central to the cultural identity of the diverse Yao language groups, and the Yao across China use indigo dyes [17]. However, despite several studies by Chinese authors on indigo dyeing in Yunnan [18, 19], Guizhou [20], Guangxi [21], and Sichuan [22], none of these studies recorded the scientific names of the plant species that were sources of indigo. Yet, detailed records of indigo dyeing are important not only to record the past, but also for the future.

This study had two aims: Firstly, to record traditional indigo dyeing processes, by quantifying and timing indigo pigment extraction. Secondly, to document the plant species used in traditional indigo pigment extraction and subsequent dyeing processes by Landian Yao people.

**Fig. 1:**
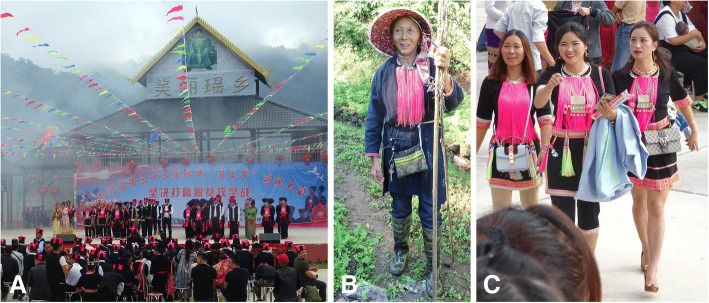
The grand celebration for “Pan Wang Festival”, and Yao people have the custom of wearing traditional garments that are dyed with indigo dyes. **a** Modern ceremony of the “Pan Wang Festival”. **b** Traditional garments of the old generation that are made from natural indigo dyes. **c** Traditional garments of the younger generation that are made from synthetic indigo dyes

To put this study in context, a brief background on terminology and the geographic distribution of the Landian Yao are important. The Landian Yao is part of the broader Hmong-Mien language family spread across southwestern and Southern China, Laos, Thailand, and Vietnam [23, 24]. In China, Landian Yao (literally “blue clothes Yao”) is a widely accepted term. In Vietnam, the name given for this language group is Dao, and in China, Landian Yao, as part of an officially recognized nationality, the Yao. In the study area, people call themselves You Mien, which is closer to the widely recognized linguistic group, Iu Mien [24]. Internationally, the Iu Mien (You Mien, highland Yao, or Landian Yao) is the largest Yao language group, with the Kim Mun, as the second largest Yao language group, with a 79% similarity between these two Yao languages [24]. Landian Yao used to farm by slash-and-burn agriculture, growing dryland rice and maize. Until the founding of the People’s Republic of China, the Landian Yao still lived in mountains with an elevation of 800 to 1500 m. These higher elevation areas were also suited to growth of several indigo-yielding plant species. Textile production activities were an essential part in traditional life of Landian Yao people. They also were an important skill of women for seeking respect and striving for the corresponding social status in Landian Yao [25]. Landian Yao’s traditional textile culture encompasses the cultivation and production of raw materials related to textiles. It is divided into the cultivation of cotton and indigo-yielding plants and the extraction of indigo dye [51]. Landian Yao has a long history of cultivating indigo plants and processing indigo dye which were used to dye their traditional cotton textiles [25], and cotton, indigo paste, undyed, and dyed cloth and traditional clothing have all become commodities among Landian Yao in exchange for daily necessities [51]. Landian Yao dyers use plastic vats for indigo dye extraction in self-sufficient mode; however, those who make indigo dye as a livelihood generally require a big pool outside their house [51]. The details of which indigo plant species were used were not previously recorded, however. Although fashions have changed, traditional textiles dyed with indigo are still important to the Landian Yao cultural identity. Each man in Landian Yao must have a religious ritual called “*dujie*” when he becomes an adult. On the ritual, he must prepare a new traditional costume and cannot wear old clothes, because “*dujie*” symbolizes the new beginning [52]. The annual post-harvest “Pan Wang Festival” (Fig. 1A), that links to their ancestor, Pan Wang, also takes place in Vietnam [23]. On this day, Yao people have the custom of wearing traditional garments that are dyed with indigo dyes, but traditional garments of the old generation are made from natural indigo dye (Fig. 1B), and the younger generation’s are all made from synthetic indigo dye (Fig. 1C), because they believe that it is only in this way that they can be identified by ancestors [26].

## Methods

### Study area

**Fig. 2:**
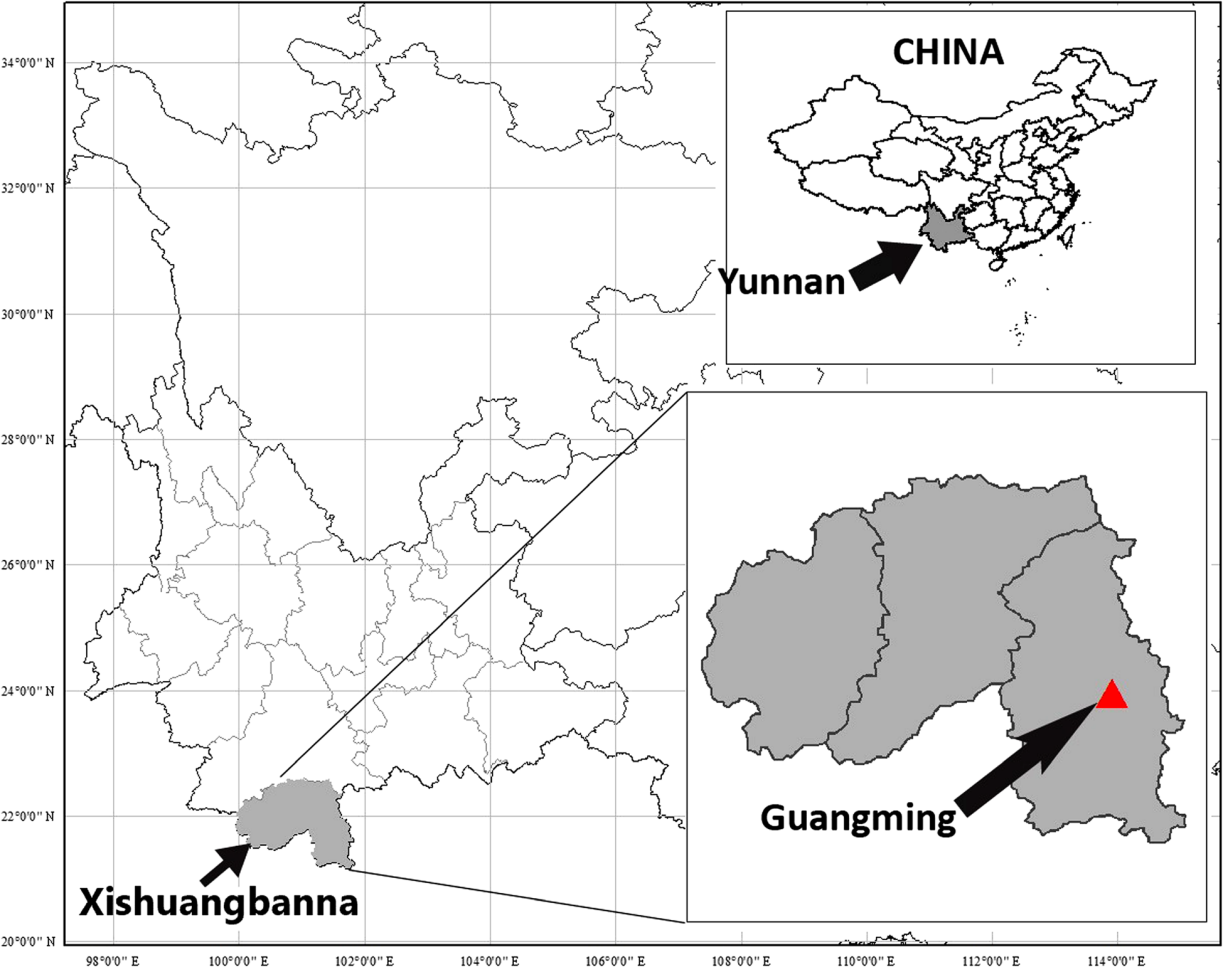
The location of Guangming Village in Southwest Yunnan, China

This study was carried out in Guangming Village, which is part of Yaoqu Township, the only Yao people inhabited town in Xishuangbanna Prefecture of Yunnan Province, China (Fig. 2). Located between 21°37′ and 21°58′ north latitude and 101°24′ and 101°35′east longitude, the main indigenous people in the town are Yao (or Mien), Dai (Thai) and Hani (Akha) [26]. Among them, the language used by “You Mien” and “Jinmen” belongs to the Mienic language branch of the Hmong-Mien language group of the Sino-Tibetan language family [17]. Guangming Village had about 300 inhabitants and all of them were the Landian Yao. In the late Ming and early Qing dynasties (1627~1684), the Yao ancestors moved from Hunan and Guangxi to Yunnan and entered the mountainous areas along the border of Mengwang Township and Mengla County in Xishuangbanna through Kaihua County, Pingbian County, Simao County, and Jiangcheng County in Yunnan Province [27]. Most of the Landian Yao in the Guangming Village are dependent on farming and income from rubber tapping. The main criteria for choosing the study site is the fact that some Landian Yao still continued to use natural indigo.

### Field research

Ethnobotanical fieldwork took place over a 40-days spread over a 3-year period between September 2015 and November 2018. We interviewed 46 key informants. Information is collected primarily through semi-structured interviews, participatory approaches, and group discussions with informants involved with indigo dyeing. Most of the key informants were elderly people. The age of key informants ranged between 36 and 82 years old. The average age of men and women is 63 and 60, respectively. The number of female dyers in the interview sample (*n* = 30) was almost twice that of male dyers (*n* = 16). The questions during the interviews were mainly on the type of indigo-yielding plant species used, related knowledge and practices, and the purpose of dyeing and its relevance to livelihood (Appendix 1). We participated in the collection of indigo-yielding plant species (*Strobilanthes cusia* (Nees) Kuntze, *Indigofera tinctorial* L. and *Indigofera suffruticosa* Mill.). Digital cameras were used to record the process of indigo extraction. We also documented the ethnobotanical information for each plant species, including scientific name, local name, parts used, and habitat. Scientific names conform to those in The Plant List (http://www.theplantlist.org). Voucher specimens of all the species listed here from 15LS04, 16LS01-16LS13, to 18LS01-18LS05 were collected and deposited at the herbarium of Kunming Institute of Botany.

### Data analysis

The use frequency of key influence factors in the indigo dye extraction was estimated by utilization frequency [28].

$$ f=\frac{N_{\mathrm{m}}}{N_{\mathrm{i}}} $$

where *N*_m_ was the number of use reports of key influence factors (such as indigo-yielding plant material to tap water, amount of lime, fermentation duration, agitation time, and precipitation time) mentioned by informants, and *N*_i_ was the number of informants. High *f* values indicated the key influence factors used frequently in the indigo dye extraction.

## Results

### Indigo-yielding plant species

During our ethnobotanical surveys, we found that three plant species were used for indigo production (Table 1). *Strobilanthes cusia* is the main indigo source and is cultivated in home gardens and agroforestry systems in the study area. Two wild folk varieties of *Strobilanthes cusia* (*gam lu* and *hong gong gam*) were also used by local dyers and were transplanted from montane forest into home gardens. Dyers consider that indigo yields and indigo quality from *Indigofera suffruticosa* and *Indigofera tinctoria* are lower than from *Strobilanthes cusia*. Consequently, these species are used by fewer and fewer people since people moved from their mountain village (Suo Shan Jiao) to Guangming 50 years ago. And even in the past 3 years, we have noticed a marked decline in the use of both of these species between our first visit to the study area (2015) and our most recent visit in November 2018.

**Table 1:** Detailed information of Indigo plant species

Family	Scientific name	Local name	Chinese name	Parts used	Source of plant material	Specimen number
Acanthaceae	*Strobilanthes cusia* (Nees) Kuntze	Gam	Ban Lan	Aerial part	Cultivated (and wild)	15LS04
Fabaceae	*Indigofera tinctoria* L.	Gam sam sei	Mu Lan	Leaves and stems	Naturalized	16LS11
Fabaceae	*Indigofera suffruticosa* Mill.	Gam sam bu	Ye Qing	Leaves and stems	Naturalized	16LS12

**Fig. 3:**
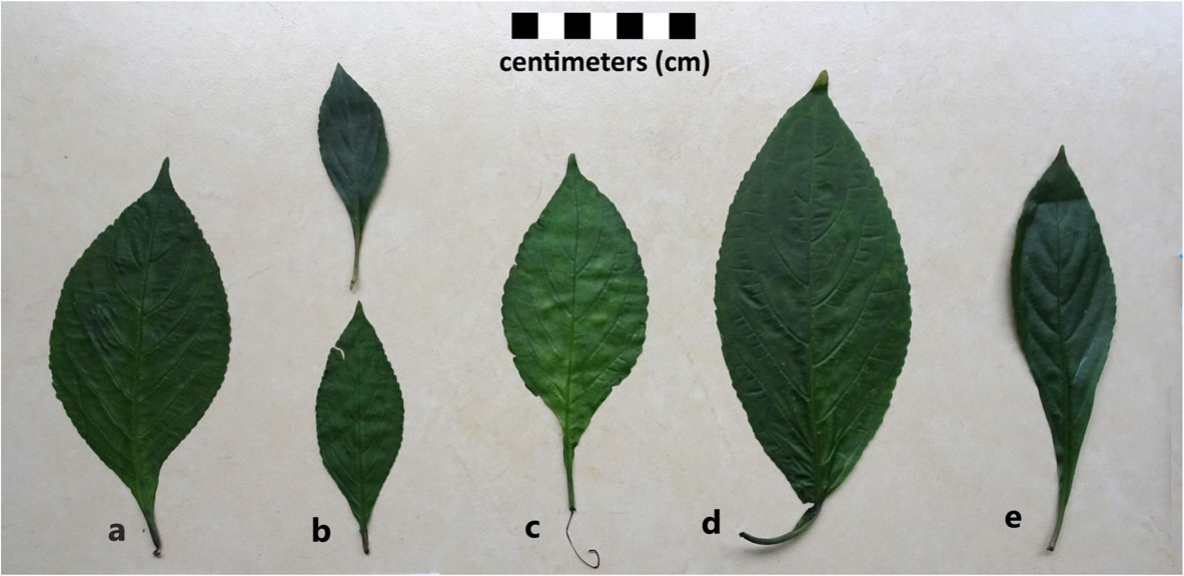
The leaves of five of the six folk varieties recognized by Landian Yao in the region. From left to right is **a** *hong gong gam*, **b** *gam sam*, **c** *gam nyoyi bu*, **d** *gam lu*, and **e** *Akha gam*

According to the growth environment and morphological differences, *Strobilanthes cusia* is divided into six varieties or phenotypes by Landian Yao in the region (Table 2). Among them, there are five cultivated types in which the local names are *gam lu, gam gai*, *gam sam, gam nyoyi bu*, and *Akha gam*, respectively. All the varieties of *Strobilanthes cusia are* called “*gam*” in the study area. *Gam sam* means the *Strobilanthes cusia* with small leaves; the meaning of “*sam*” is “small”. Compared with *gam sam*, the leaves of *gam gai* are larger. Among the three, *gam lu* has the largest leaves; the meaning of “*lu*” is “big”. *Gam nyoyi bu* has thicker leaves than *gam lu*, and its nodes are covered by gray hairs; “*nyoyi*” means node of stem in Yao language, and “*bu*” means the gray color. *Akha gam* means this variety is from the Akha people of Honghe in Yunnan. There is also a wild type called *hong gong gam* or *gam gong gam*. According to legend, it was planted by Pan Wang, who is widely recognized as the ancestor of Yao people, including in China and Vietnam [23]. Local dyers considered that the dyeing quality of *hong gong gam* is the best of all the *Strobilanthes cusia* varieties. These six types of *Strobilanthes cusia* can be difficult to distinguish from plant morphology, and there was some local disagreement about which folk varieties were which. In most cases, local people judged different folk varieties of *Strobilanthes cusia* on the basis of the size and shape of the leaves (Fig. 3).

**Table 2:** Detailed information of six varieties of *Strobilanthes cusia*

Local name	Meaning of name	Production types	Flowering time	Growing place	Specimen number
*Hong gong gam/gam gong gam*	It is planted by their ancestor	Seed	Flowered every year after grow up	Shade	18LS03
*Gam lu*	Big size	Cutting	Flowered every year after grow up	Shade	18LS01
*Gam gai*	Medium size	Cutting		Everywhere	
*Gam sam*	Small size	Cutting	Every year		18LS04
*Gam nyoyi bu*	Gray node	Cutting	Every year	Shade	18LS02
*Akha gam*	It is from Akha people	Cutting	Every year	Everywhere	18LS05

### Traditional indigo pigment extraction

At the study sites, the men take charge of indigo pigment extraction. This is entailed several steps and took between 3 and 5 days. In the past, Landian Yao produced indigo for traditional clothing twice a year, with production times chosen according to the lunar calendar. The first indigo production period equated to June to August, and the second time was the lunar equivalent of September to October. However, with changes in traditional lifestyle, Landian Yao dyers now produce indigo just once a year, solely in the calendar equivalent of September to October.

*Step 1*. Fresh leaves and stems are harvested from the indigo-yielding plant species by cutting 10–12 cm from the ground. These are piled up in a large plastic vat. The cut stems and leaves are then covered with tap water and soaked in the water for 2–3 days. Heavy stones are placed on the top of the leaves and the tank to keep them in an anaerobic underwater environment. Landian Yao dyers consider that the fermentation process is completed when the stems and leaves are soft and have turned black. During the fermentation, in order to make sure all the leaves are soaked and fermented, the vat is agitated up and down once a day with a wooden rake.

**Fig. 4:**
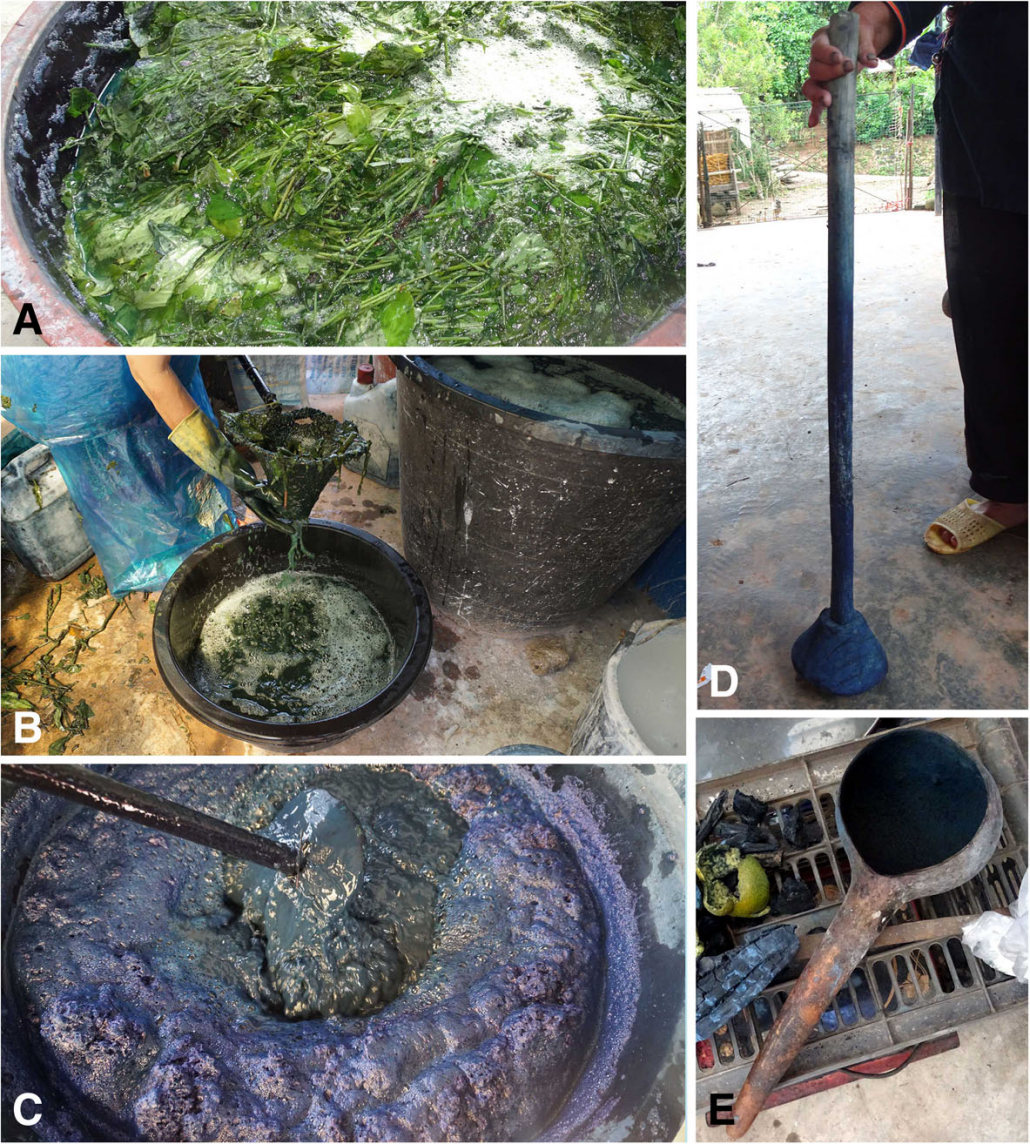
The indigo paste production process. **a** *Strobilanthes cusia* leaves after 24 h of fermentation. **b** Removal of leaves using a net (called *jiu* in local name). **c** Oxygenation after adding lime water to reduce the pH. **d** Detail of wooden tool (*dongzhong*) used for oxygenation. **e** A half section of a gourd (*Lagenaria siceraria*) scoop used to take out indigo paste at the end of the process

*Step 2*. After the fermentation is finished, the liquid has turned blue-green (Fig. 4A). The plant residues are drained and removed from the vat with a net (Fig. 4B). Lime powder is then mixed in water in a small container then gently added to the liquid. At this stage, the amount of lime depends on the experience of the indigo producer which is generally judged by the color of the foam created by stirring the mixture. A few people also taste the water in the vat. The liquid is then beaten vigorously with the wooden tool (*dongzhong*) (Fig. 4D) to oxygenate the liquid in the vat (Fig. 4C). If the amount of lime is suitable, the froths produced by beating are red and blue and the flavor of the water tastes slightly spicy.

*Step 3*. While the froths created by beating become less, the water turns blue which is the signal to stop beating. When beating is stopped, the suspension is placed in the large plastic vat for several hours to make it precipitate, the indigo paste slowly sinks to the bottom. After the indigo paste precipitated to the bottom, remove the supernatant from the top with a bowl carefully. The paste is then collected (Fig. 4E) by pouring it into a container, and maintain the state of the indigo paste by adding water regularly.

The detailed recording for indigo pigment extraction by Landian Yao (Appendix 2) evaluated by the ethnobotanical quantitative method concluded that the ratio of material to tap water with the highest utilization frequency is 30 kg: 200 l (lime 3 kg), in which the utilization frequency index is 0.370. The fermentation duration with the highest utilization frequency is 2–3 d, in which the utilization frequency is 0.587. The mixing time with the highest utilization frequency is 60 min, in which the utilization frequency index is 0.717. The most commonly used indigo pigment precipitation time was 2–3 h, in which the utilization frequency is 0.457. So the best traditional recipe of indigo dye extraction of the Landian Yao was a ratio of *Strobilanthes cusia* material to liquid (30 kg (fresh weight): 200 l), lime (3 kg), fermentation time (2–3 days), stirring time (1 h), and settling out of the indigo pigment, 2–3 h.

### Traditional dyeing process

**Fig. 5:**
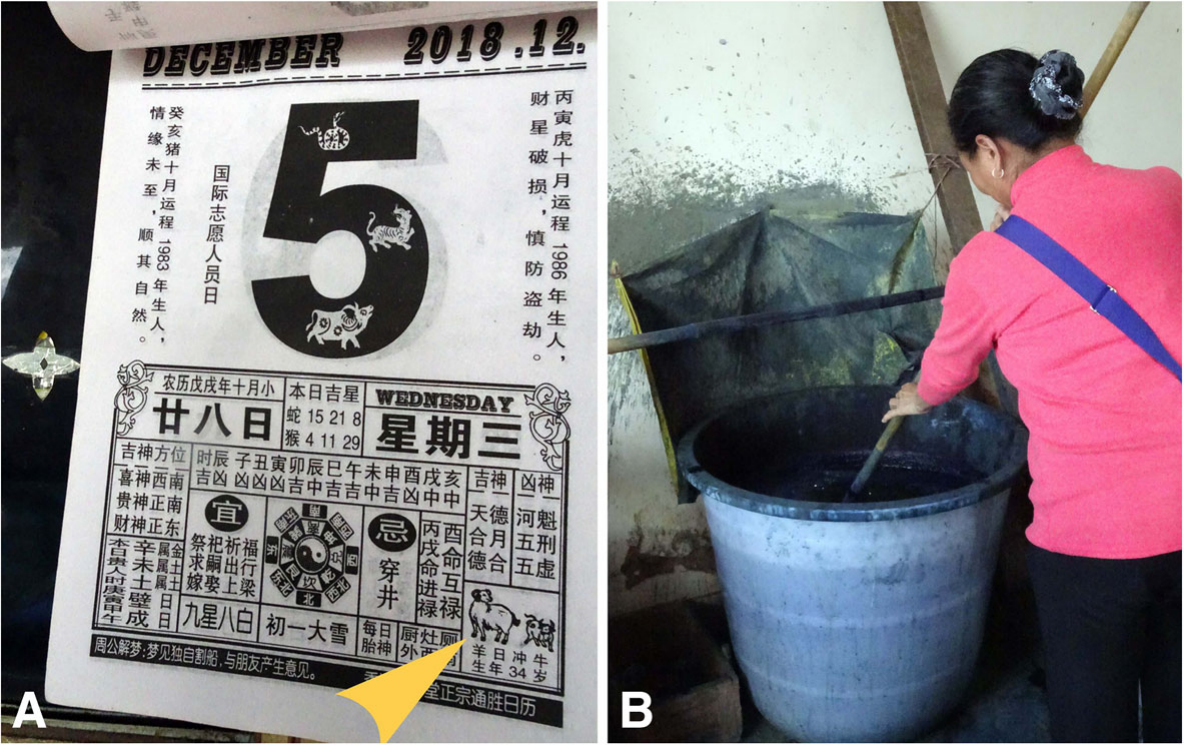
A woman was preparing the dye solutions in her house in the study area on goat day. **a** A lunar calendar used by Landian Yao people to number the days, and the arrow pointed to goat day. **b** A woman was preparing the dye solutions in her house

Women are responsible for dyeing cloth in the study site. The traditional dyeing process is only carried on the goat days from June to October in the lunar calendar. Landian Yao people number the days according to a lunar calendar (Fig. 5A). They also use the Chinese zodiac to represent different days. The rat, ox, tiger, rabbit, chinese dragon, snake, horse, goat, monkey, rooster, dog, and pig are the 12 symbolic animals of the Chinese zodiac. Every day has a symbolic animal for 12 days per one round. Indigo paste is added to the plant water, together with maize (*Zea mays* L.) wine, ash water, and the hot maize or chillies (*Capsicum annuum* L.). Leaves and stems of *Tithonia diversifolia* (Hemsl.) A.Gray and *Peristrophe bivalvis* (L.) Merr. are boiled in the water with the stems and leaves of *Buddleja officinalis* Maxim and *Persicaria hydropiper* (L.) Delarbre. Dyers then collect plant water by filtration.

Ash water is the filtrates of wood ash from *Bauhinia variegata* L. or the burning ashes from *Artemisia argyi* or rice straw (from *Oryza sativa* L.). To get darker reddish textiles, Landian Yao dyers add water from boiling the stems and leaves of *Iresine herbstii* Hook*.*, an introduced species from South America. The dye vat that was used took about a week to ferment, until the color of the dye liquid turned to yellow-green. Use a wooden stick to stir the dye liquid, the bubble is bright, and the dye liquor taste a little sweet and a little spicy. When all of the above conditions are satisfied, the dyeing solution can be used to dye cloth. The dyeing vat can be maintained in a functional state for many months. If there is no such phenomenon emerged in the dye liquor almost 1 week later, add the water from boiling the whole plants of *Ageratum conyzoides* (L.) L. or the leaves of wild *Piper bavinum* C. DC. to accelerate the fermentation process, and then wait for 1 to 2 days. If the color of the dyeing vat is bright yellow, it means strong alkalinity and more indigo paste should be added. If the color is dark blue, it means alkalinity is not enough and needs more ash water.

The woven cloth first needs to be wet so that the pigment attaches well to the cotton fibers. Dyers usually place the cloth in the dyeing solution for 1 h, and then take out to wash and dry it. They used to dye textiles blue by multiple dips (at least 10 times) (Fig. 5B).

**Fig. 6:**
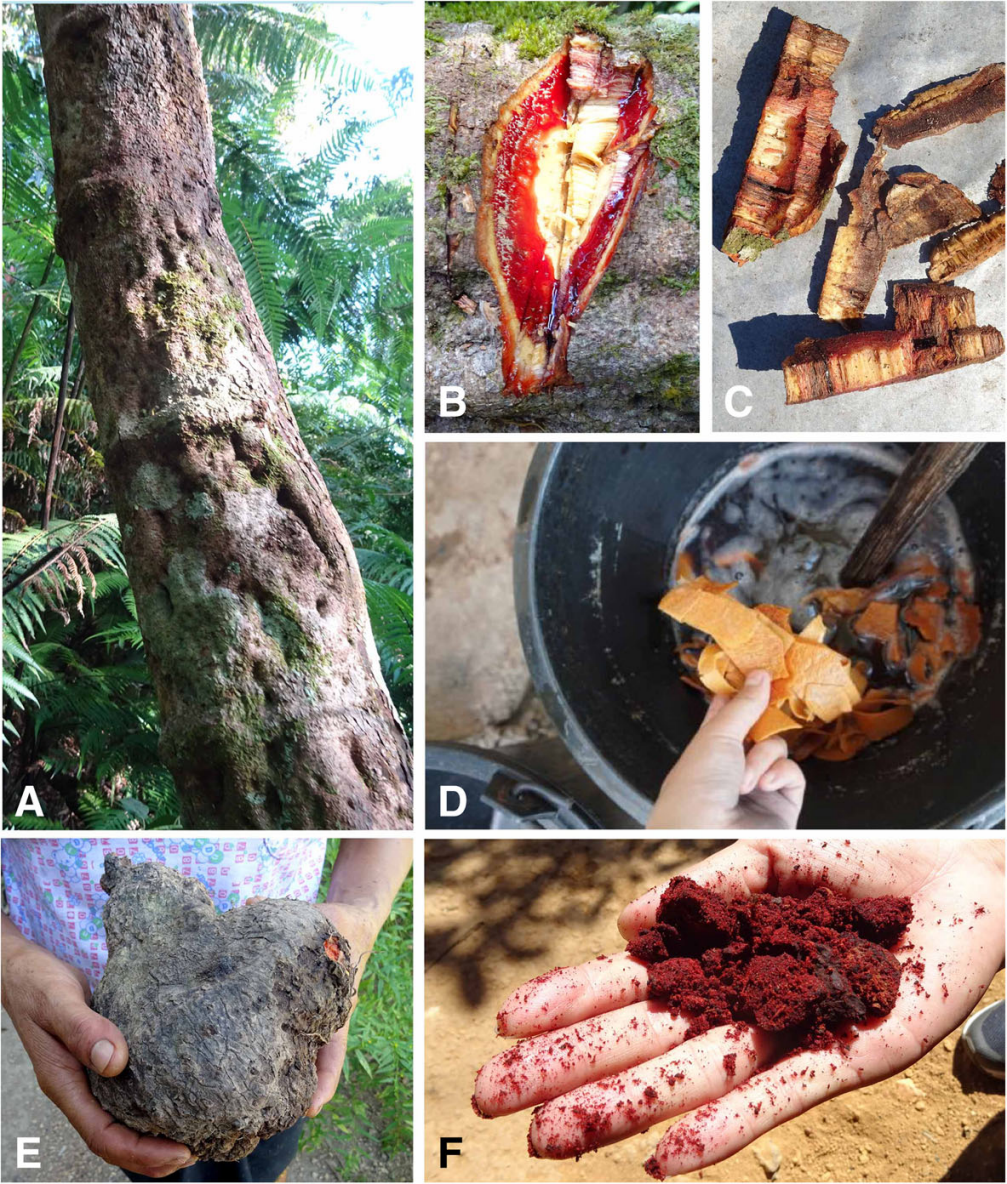
Tannin-rich species added to the second dyeing solution. **a** The large (15-cm diameter) stem of the forest liana *Spatholobus suberectus.* **b** Cut stem showing characteristic red exudate. **c** Cut sections of *Spatholobus suberectus* stem. **d** Sliced tubers of *Dioscorea cirrhosa* soaking in the water. **e** Residue of *Dioscorea cirrhosa* after dyeing. **f** Wild harvested *Dioscorea cirrhosa* tuber

The second dyeing step (Fig. 6 E, F) is production of an aqueous solution from the sliced tubers of *Dioscorea cirrhosa* or the cut stems of the forest liana *Spatholobus suberectus* Dunn (Fig. 6 A–D). Traditionally, the dyed blue cloth is put in the second dyeing solution for half an hour, and taken out and beaten with a hardwood stick, then put in the tannin-rich solution again. This is repeated two to three times, then the cloth is rinsed and hung up to dry. Color differences are obvious that the cloth is soaked in the second dyeing solution or not. The color of the cloth soaked in the second solution is black.

During the dyeing process, in addition to the indigo blue dyes, other plants were introduced into the dye vat. Our ethnobotanical surveys documented 14 plant species, belong to 11 families, used in this part of the dyeing process (Table 3). Only *Dioscorea cirrhosa* and *Artemisia argyi* were recorded but other species were first reportedly used in the indigo dyeing process by this study.

**Table 3:** Plant species used by Landian Yao in addition to indigo source species during the dyeing process

Order	Family	Scientific name	Local name	Chinese name	Parts used	Source of plant material	Usage	Specimen number
1	Asteraceae	*Ageratum conyzoides* (L.) L.*	Masei	Huo Xiang Ji Shu	Whole plant	Naturalized	Fermentation source	16LS03
2	Asteraceae	*Artemisia argyi* H.Lév. & Vaniot	Mengwei	Ai Hao	Leaf	Wild	Ash source	16LS01
3	Amaranthaceae	*Iresine herbstii* Hook.*	Gamsei	Xue Xian	Leaf and stem	Cultivated	Symbolic reason	16LS02
4	Acanthaceae	*Peristrophe bivalvis* (L.) Merr.	Nanlamu	Guan Yin Cao	Leaf and stem	Cultivated	Fermentation source	16LS09
5	Asteraceae	*Tithonia diversifolia* (Hemsl.) A.Gray*	Mafangwang	Zhong Bing Ju	Leaf and stem	Naturalized	Fermentation source	16LS04
6	Dioscoreaceae	*Dioscorea cirrhosa* Lour.	Dongyang	Shu Liang	Tuber	Wild	Tannin source	16LS05
7	Fabaceae	*Bauhinia variegata* L.	Moonbulouyang	Fen Hua Yang Ti Jia	Stem	Wild	Ash source	16LS06
8	Fabaceae	*Spatholobus suberectus* Dunn	Zhengzhongmei	Mi Hua Dou	Bark	Wild	Tannin source	16LS10
9	Gramineae	*Oryza sativa* L.	Bugao	Dao	Stem	Cultivated	Ash source	
10	Loganiaceae	*Buddleja officinalis* Maxim.	Wonggongmagia	Mi Meng Hua	Whole plant	Wild also cultivated	Fermentation source	16LS13
11	Piperaceae	*Piper bavinum* C. DC.	Lao	Hu Jiao Shu	Leaf	Wild	Fermentation source	16LS07
12	Polygonaceae	*Persicaria hydropiper* (L.) Delarbre	Meliu	Shui Liao	Whole plant	Cultivated	Fermentation source	16LS08
13	Poaceae	*Zea mays* L.*	Baogu	Yu Shu Ji	Rachis	Cultivated		P1601
14	Solanaceae	*Capsicum annuum* L.*	Longma	La Jiao	Dry fruit	Cultivated		P1602

Species in inventory are arranged firstly by family taxa and then by genus taxa. Voucher number with 16LS means voucher specimen number, and the one with P16 means voucher photograph number. All the specimens of documented species were collected and deposited at the herbarium of Kunming Institute of Botany

*introduced species, all of which are from Latin America.

## Discussion

### The Landian Yao dyers

There are 46 key informants in this study in which the number of females is almost twice to that of males. In the study area, men usually take charge of indigo dye extraction, while women are responsible for dyeing cloth in the study sites. This agrees with reports from Landian Yao people of Yuanyang County in Yunnan Province [51] By contrast, indigo dyeing in Indonesia and Timor-Leste [29] and northeastern Thailand is mostly held by women [30]. In these cases, indigo production is considered to be ritually polluting to men and traditional dye studios are away from the main household [29]. The difference with East Timor, Indonesia, and Thailand may be because the particularity of indigo dyeing by the Landian Yao. Firstly, indigo dye extraction is a very taxing work and unlike these other parts of Asia, Landian Yao men are involved in vigorous stirring of the indigo extraction vat. Secondly, selling of the indigo paste was an economic mainstay in the past, with indigo extraction done on a much larger scale in the study area than in East Timor or Indonesia [29, 31, 32].

### The indigo-yielding plant species

In contrast to this study, an earlier report on the traditional textile dyeing technology of Yao communities in Wuling (which refer to three provinces in China’s southern Hunan, Jiangxi, Guangdong and Guangxi mountains collectively) showed that indigo plants included *Strobilanthes cusia*, *Indigofera tinctoria*, and *Persicaria tinctoria* (Aiton) H.Gross [33]. Indigo-yielding plant species in Dong communities (in Hunan, China) from which the blue dye is extracted were either from *Strobilanthes cusia* or *Persicaria tinctoria* [34]. *Strobilanthes cusia* is widely used as an indigo-yielding plant species and has the most commercial potential due to its high indigotin levels compared to another popular indigo source, *Indigofera tinctoria* [35]. Three folk varieties of *Strobilanthes cusia* which can be considered as folk (cultural) varieties were found in the ethnobotanical studies on dye plants used in Xishuangbanna [36]. In this study, six folk varieties of *Strobilanthes cusia* were named by local dyers. These were distinguished primarily on the basis of leaf size and shape, leaf thickness, habitat, and, for some folk varieties, the color of the nodes. This higher number of *Strobilanthes cusia* folk varieties used and cultivated in the study area suggest that *Strobilanthes cusia* has a high cultural value for Landian Yao people. It also raises the question of whether these folk varieties are genetically different or whether their different characteristics are a function of soil fertility and different habitats (e.g, shaded home gardens, cultivation in sunny garden sites, or montane forests). Selection of different varieties of culturally important species from wild sources is well known from studies of edible plants such as taro (*Colocasia esculenta* (L). Schott) in China [37] or *Leucaena esculenta* (DC.) Benth. (Fabaceae) and *Stenocereus stellatus* (Pfeiff.) Riccob. (Cactaceae) in Mexico [38, 39]. But cultural selection of indigo-yielding plant species has not been studied. In the study area, discussions with local Landian Yao showed their detailed knowledge of sites that were suitable or unsuitable for *Strobilanthes cusia* cultivation, both at a landscape level and within home-gardens. Our field observation of leaf characteristics (size, color) and previous studies of the fertility requirements of *Strobilanthes cusia* [40] suggest that soil fertility and habitat may determine *Strobilanthes cusia* characteristics as much as the genetics of variety selections. Shading through inter-cropping is also known to influence *Strobilanthes cusia* growth form [41]. Consequently, we suggest further study on the genetics, fertility requirements, and habitat influences that may determine folk varieties of *Strobilanthes cusia*.

### The indigo dye extraction procedures

The general procedures of indigo dye extraction are similar as other Landian Yao villages in Guangxi, of which there are only two differences. Firstly, Landian Yao villages in Guangxi build large pools for indigo dye extraction. Secondly, the ratio of fresh plants to lime is different. This may be because the purity of lime is not the same, leading to a difference in the alkalinity of lime water [53].The principle of indigo dye extraction is as follows: The precursor of indigo in the indigo plants is a colorless glucoside which is referred to indican; During the fermentation, indican in the leaves or stems is hydrolyzed by enzyme to indoxyl; Subsequently, indoxyl is oxidized to form indigo under alkaline conditions [42]. The enzyme is derived from fresh indigo plants which release the glycolytic enzyme from the plant cells through maceration [35]. The alkaline conditions are provided by adding lime. The lime could also combine with carbon dioxide produced by fermentation to generate calcium carbonate with adsorption to precipitate indigo [43]. The stirring time is equally important so that as much oxygen as possible comes into contact with the indoxyl. There are numerous empirical factors involved in the traditional indigo dye extraction method. However, we can find some key influence factors in indigo dye extraction combined with scientific principles. The key influence factors include the ratio of material to water, pH (the amount of lime), and fermentation processes. A better understanding of these processes can lead to improvement in the traditional indigo dye extraction method so as to raise the indigo dye production or improving quality.

### The theory of indigo dyeing process

The local people are only dyeing cloth on goat days. Under our investigation, this selection is not related in any obvious way to the environmental conditions, climate change or something else. However, the goats are black in the study site. According to the local residents, they think it looks good to wear a dark color. A deep color of clothing is a symbol of wealth. They choose the goat days if they can get a deep color like the color of the goats through indigo dyeing which should be a superstition.

Typically, the color of indigo-dyed cloth temporarily appeared green when first taken out from the dyeing vat. Then, when exposed to air, the green color slowly changed to blue on oxydization. This phenomenon could be explained by the underlying chemical principle of the dyeing process. Indigo dye is a water-insoluble pigment. Therefore, the indigo molecules must be reduced to leuco-indigo which is colorless and dissolves in the water before they can penetrate into the fabrics and be absorbed by the fibers in the dyeing process. The fabrics are then oxidized in the air and the leuco-indigo molecules become indigo blue molecules again. This leuco-indigo is white, but it is yellowish-green in reality [44].

### The roles of adding plant species during the dyeing process

The first stage of the dyeing process is to dissolve and reduce the insoluble dyestuff in a warm alkaline solution. The reducing agent for the reduction of indigo blue molecules to leuco-indigo molecules is mainly from the hydrogen produced during the fermented indigo dyeing vat [45]. To increase the temperature of the dye vat, a hot mixture of whole maize (*Zea mays*) cobs and whole chillies (*Capsicum annuum*) is added by Landian Yao to the indigo dye vat. The alkalinity of dyeing liquid is increased by adding of ash water (lye) [47]. The ash for the dye is obtained from a variety of plant species included rice straw (*Oryza sativa*), *Bauhinia* *variegata*, and *Artemisia argyi* or alternatively, ash from cooking fires. The nutrients and microorganisms required for the fermentation of the dyeing liquid are probably derived from the monosaccharides or polysaccharides hydrolyzed by the tissues of the plants [46]. *Buddleja officinalis*, *Tithonia diversifolia*, *Peristrophe bivalvis*, and *Persicaria hydropiper* may be sources of these nutrients and microorganisms that contribute to fermentation. The scientific basis for Landian Yao dyers adding *Ageratum conzyzoides* and *Piper bavinum* to possibly accelerate the fermentation is still unknown. *Dioscorea cirrhosa* and *Spatholobus suberectus* are tannin mordants which have three effects in blue dyeing. First, the dyes from these two spcies react with indigo blue to become a culturally desired black color. Second, the addition of these tannin-rich species appears to improve color fastness. Third, these two species may compensate for the loss of the degumming quality of the fabric in multiple dyeing processes so that the fabrics have washing endurance [48–50]. The local people also place value on the symbolic potency of red color. The reddish-purple color of *Iresine herbstii* leaves may account for use of this introduced South American ornamental species being added to indigo in the textile dye process. Other Landian Yao villages in Guangxi are using several different plant species during the dyeing process and they have to put the cloth into the cow skin water [53].

## Conclusions

In the Landian Yao (Iu Mien) area, traditional costumes and their production techniques are skills that were widespread due to the link between traditional textiles and cultural identity. Indigo dyeing is a complex process. In the study area, people use several previously unrecorded sources of high pH ash water (lye) as well as tannins (from *Dioscorea cirrhosa* and *Spatholobus suberectus*) and different plant species to enhance fermentation. *Strobilanthes cusia* is the most popular source of indigo pigment and this is reflected both in its widespread use and in recognition of six different folk varieties of *Strobilanthes cusia*. Historically, indigo dyeing was only done on goat days twice a year according to the lunar calendar. Over the past two decades, the frequency of indigo dyeing and its associated knowledge has faded due to changing values and the fashion sense of the younger generation. Moreover, the current scale of traditional indigo dyeing is not suitable for modern and rapid industrial production. Nevertheless, Landian Yao knowledge and practices of indigo extraction and dyeing are part of China’s intangible cultural heritage. In this regard, this study has recorded detailed information on traditional indigo dyeing as part of this cultural heritage. Considering the human environmental health issues related to the synthetic indigo, natural indigo can be a good choice which could be reintroduced into the market. But this requires indigo producers to get sufficient returns for what is a labor-intensive process. If this occurs, then traditional indigo dyeing can significantly contribute to local economic activity and cultural traditions.

## Appendix 1

### Outline of a semi-structured interview

1. What is your age?

2. When do you use indigo dyes?

3. What are the occasion and the specific usage?

4. What is the local name of indigo plant and its meaning?

5. What is the habitat, flowering time and planting methods of each indigo plant?

6. Which do you think is the best quality of indigo plant species?

7. What is the indigo dye preparation method and who complete it?

8. What is the traditional dyeing method and who finish it?

9. What are common used parts?

10. What is the function of each plant in the dyeing process?

11. Which ash species providing the alkaline environment in the dyeing process?

## Appendix 2

**Table 4:** Detailed recording for traditional indigo dye extraction by Landian Yao

Informant number	Material to tap water (kg:l)	Lime (kg)	Fermentation duration (d)	Mixing time (min)	Stewing time (h)
1	30:150	3	2–3 (hot), 3–4 (cool)^*^	10–20	3–4
2	50:150	5	3–4	60	2–3
3	30:150	3	2–3	60	2
4	60:200	2.5	3–4	60	5–6
5	40:150	1.5	2–3	60	1–2
6	40:150	1.5	2–3 (hot), 3–4 (cool)	60	2
7	30:200	1.5	2–3 (hot), 7–8 (cool)	30	5–6
8	25:150	2	2–3	60	12
9	25:150	1	2–3	60	2
10	35:200	1.5	3–4	30	2
11	35:200	5	2–3	60	12
12	30:200	3	3–4	60	2–3
13	30:200	3	2–3	60	2–3
14	30:200	3	3–4	60	2–3
15	25:150	3	2–3	30	5–6
16	30:100	1	2–3	15–20	12
17	40:150	1.5	2–3	60	2–3
18	35:200	3	3–4	60	5–6
19	30:150	3	2–3	60	2–3
20	20:150	2	2–3	60	2–3
21	30:200	3	2–3	60	5–6
22	30:150	3	3–4	30	1–2
23	35:200	3	2–3	60	2
24	50:250	2	3	30	12
25	30:200	3	2–3	30	2–3
26	25:150	2	2–3	30	2–3
27	25:200	3	2–3	60	2–3
28	30:200	3	2–3	60	2–3
29	30:200	3	2–3	60	2–3
30	25:150	2	2–3	60	1–2
31	30:200	3	2–3	60	2
32	30:200	3	2–3	60	1–2
33	25:150	2.5	2–3	30	2–3
34	30:200	3	2–3	30	2–3
35	30:200	3	3–4	60	5–6
36	25:200	3	3–4	60	4–5
37	30:200	3	2–3	60	2–3
38	30:200	3	2–3	10–20	3–4
39	25:200	2.5	2–3	60	2–3
40	30:200	3	3–4	60	2–3
41	30:200	2.5	3–4	60	3–4
42	30:200	3	2–3 (hot), 5–6 (cool)	60	2–3
43	30:200	3	2–3 (hot), 3–4 (cool)	30	2–3
44	25:200	2.5	2–3 (hot), 3–4 (cool)	60	2–3
45	25:150	2	2–3 (hot), 3–4 (cool)	60	2–3
46	30:200	3	2–3	60	3–4

*fermentation time required in the hot or cool days
